# Soil N_2_O and CH_4_ emissions from fodder maize production with and without riparian buffer strips of differing vegetation

**DOI:** 10.1007/s11104-022-05426-0

**Published:** 2022-04-11

**Authors:** Jerry C. Dlamini, L. M. Cardenas, E. H. Tesfamariam, R. M. Dunn, J. Evans, J. M. B. Hawkins, M. S. A. Blackwell, A. L. Collins

**Affiliations:** 1 Department of Soil, Crop and Climate Sciences, University of the Free State, 9300 Bloemfontein, South Africa; 2 Sustainable Agriculture Sciences, Rothamsted Research, North Wyke, Okehampton, Devon EX20 2SB UK; 3 Department of Plant and Soil Sciences, University of Pretoria, Private Bag X20, Hatfield, 0028 South Africa; 4 Computational and Analytical Sciences, Rothamsted Research, West Common, Harpenden, Hertfordshire AL5 2JQ UK

**Keywords:** Nitrous oxide, Methane, Maize, Vegetated riparian buffer strips

## Abstract

**Purpose:**

Nitrous oxide (N_2_O) and methane (CH_4_) are some of the most important greenhouse gases in the atmosphere of the 21st century. Vegetated riparian buffers are primarily implemented for their water quality functions in agroecosystems. Their location in agricultural landscapes allows them to intercept and process pollutants from adjacent agricultural land. They recycle organic matter, which increases soil carbon (C), intercept nitrogen (N)-rich runoff from adjacent croplands, and are seasonally anoxic. Thus processes producing environmentally harmful gases including N_2_O and CH_4_ are promoted. Against this context, the study quantified atmospheric losses between a cropland and vegetated riparian buffers that serve it.

**Methods:**

Environmental variables and simultaneous N_2_O and CH_4_ emissions were measured for a 6-month period in a replicated plot-scale facility comprising maize (*Zea mays* L.). A static chamber was used to measure gas emissions. The cropping was served by three vegetated riparian buffers, namely: (i) grass riparian buffer; (ii) willow riparian buffer and; (iii) woodland riparian buffer, which were compared with a no-buffer control.

**Results:**

The no-buffer control generated the largest cumulative N_2_O emissions of 18.9 kg ha^− 1^ (95% confidence interval: 0.5–63.6) whilst the maize crop upslope generated the largest cumulative CH_4_ emissions (5.1 ± 0.88 kg ha^− 1^). Soil N_2_O and CH_4_-based global warming potential (GWP) were lower in the willow (1223.5 ± 362.0 and 134.7 ± 74.0 kg CO_2_-eq. ha^− 1^ year^− 1^, respectively) and woodland (1771.3 ± 800.5 and 3.4 ± 35.9 kg CO_2_-eq. ha^− 1^ year^− 1^, respectively) riparian buffers.

**Conclusions:**

Our results suggest that in maize production and where no riparian buffer vegetation is introduced for water quality purposes (no buffer control), atmospheric CH_4_ and N_2_O concerns may result.

## Introduction

Nitrous oxide (N_2_O) and methane (CH_4_) are important greenhouse gases that contribute more than 21% of radiative forcing in the greenhouse effect (IPCC 2014). Although N_2_O and CH_4_ are less abundant than carbon dioxide (CO_2_) in the atmosphere, their respective global warming potentials (GWP) over a 100-year period are respectively ~ 310 and ~ 28 times that of CO_2_ (IPCC 2014; Ramaswamy et al. 2001). Soils play a vital role in N_2_O and CH_4_ regulation (Conrad 2007; Firestone 1982; IPCC 2008). Soils of natural and semi-natural agroecosystems, including croplands, grasslands, and forests, are sources or sinks of N_2_O and CH_4_ and thus play a significant role in balancing their atmospheric concentrations (Dutaur and Verchot 2007; Smith et al. 2000; Stehfest and Bouwman 2006).

In soils, N_2_O and CH_4_ are produced or consumed as a result of microbial processes (Ball et al. 1999; Conrad 2007; Yao et al. 2017). N_2_O is predominantly produced as a by-product of two microbial processes; nitrification and denitrification (Bowden 1986; Davidson 2009). In the case of CH_4_, production occurs due to organic material decomposition by methanogens under anaerobic conditions in soils (Smith et al. 2018b; Yamulki and Jarvis 2002). Under such conditions and some aerobic conditions, atmospheric CH_4_ diffusing into the topsoil can be oxidized by methanotrophs, which subsequently results in CO_2_ (Jacinthe et al. 2015; Le Mer and Roger 2001).

Agronomic management practices associated with annual row crops may result in soil disturbances that affect soil microbial communities (Friedel et al. 1996), physical properties (Gronle et al. 2015), chemical properties (Neugschwandtner et al. 2014; Wang et al. 2008), temperature (Shen et al. 2018), and moisture content (Ouattara et al. 2006). The previous changes in agricultural land often result in substantial soil and nutrient runoff losses (Bechmann and Bøe 2021; Ulén 1997), including, where they are implemented, into riparian buffer strips. Riparian buffers are primarily implemented between freshwater bodies and upland agricultural land to intercept and process non-point source pollutants, including nitrates (NO_3_^−^) sourced from adjacent agricultural land (Groffman et al. 1998; Hill 1990). Their unique location in agrosystems allow riparian buffers to process non-point source pollutants through a range of processes including nitrogen (N) mineralization, N-uptake, leaching, gaseous N emissions (nitrification and denitrification) (Firestone 1982; Müller et al. 2004; Reinsch et al. 2018), CH_4_ oxidation, and methanogenesis (Le Mer and Roger 2001; Luo et al. 2013; Megonigal and Guenther 2008). These processes are responsible for N_2_O and CH_4_ production and/or uptake as well as subsequent exchanges between the soil and atmosphere. Vegetated riparian buffers recycle organic matter through their litter with further increases of soil organic carbon (C) (Tufekcioglu et al. 2001), and seasonal moisture from high soil water tables. When the elevated soil C and high soil moisture in riparian buffers come into contact with NO_3_^−^-rich sediments intercepted from agricultural lands, N_2_O-producing processes including denitrification are promoted (Choi et al. 2006; Garcia and Tiedje 1982). Both soil C and NO_3_^−^ are energy sources for microbial process (Beauchamp et al. 1989). Anaerobic conditions and elevated soil C prevailing in riparian buffer areas can further increase processes that produce CH_4_ (Dlamini et al. 2022; Megonigal & Guenther 2008; Wang et al. 2017). Previous studies on N_2_O (Jacinthe et al. 2012) and CH_4_ (Mander et al. 2008) emissions from riparian buffers focused on buffer vegetation type and soil and environmental drivers of these gases. For instance, Jacinthe et al. (2012) observed larger N_2_O emissions in forested riparian buffers compared to grassed sites. Mander et al. (2008), however, found that forested riparian buffers were CH_4_ sinks.

Despite previous work, understanding N and C trace gas fluxes from adjacent cropped land compared to fluxes from riparian buffer strips remain limited. Therefore, this study evaluated the unintended emissions of N_2_O and CH_4_ from maize production from both buffered and un-buffered downslopes. The specific objectives of the study were (i) to understand the soil and environmental controls of soil N_2_O and CH_4_ in upslope maize and downslope riparian buffers with varying vegetation, and (ii) to understand whether specific riparian buffer vegetations emitted less N_2_O and CH_4_ when introduced for water quality purposes in maize production.

## Materials and methods

### Experimental site

The replicated plots used in this experiment are located at Rothamsted Research, North Wyke, Devon, United Kingdom (50°46 × 10´´N, 3° 54 × 05´´E). The area is situated at an altitude of 177 m above sea level, has a 37-year (from 1982 to 2018) mean annual precipitation (MAP) of 1033 mm (with the majority of rainfall received between October and November of each year) and mean annual temperature (MAT) of 10.1 °C (Orr et al. 2016). The experimental area has a slope of 8° and is on soils of the Hallsworth series (Clayden and Hollis 1985), or a dystric gleysol (FAO 2006), with a stony clay loam topsoil comprising 15.7% sand, 47.7% clay and 36.6% silt (Armstrong and Garwood 1991) overlying a mottled stony clay, derived from Carboniferous Culm rocks. The subsoil is impermeable to water and is seasonally waterlogged; most excess water moves by surface and sub-surface lateral flow across the clay layer (Orr et al. 2016), thereby making replicated experimental work using hydrologically-isolated plots feasible.

### Experimental design and treatments

#### Experimental set-up

The experiment was laid out as three blocks of four plots corresponding to four treatments each described in detail in Section 2.2.2 and further detail in Dlamini et al. (2022). The cropped upslope area was previously managed as a silage crop, with a permanent pasture dominated by ryegrass (*Lolium perenne* L.), Yorkshire fog (*Holcus lanatus* L.) and creeping bentgrass (*Agrostis stolonifera* L.) planted in 2016, which was ripped and ploughed on the 14th of May 2019 in preparation to plant maize and the riparian buffer areas remained untouched. Maize (*Zea mays* L.) was planted on the 17th of May 2019 for the experiment. Slurry was applied before ploughing using a slurry spreader fitted with a centrifugal pump and an injector, which supplied N, phosphorus (P), and potassium (K) at respective rates of 20.8, 12, and 46 kg ha^− 1^. Inorganic fertilizer was applied using a fertilizer spreader to ensure equal coverage at planting as N (Nitram-Ammonium nitrate), P (triple superphosphate; P_2_O_5_), and K (muriate of potash; K_2_O) at respective rates of 100, 85, and 205 kg ha^− 1^ (Table 1). During fertilizer application and planting, static chambers were removed from the maize fields and positioned at exactly the same place using a hand-held geographical positioning system (GPS; Trimble, California, USA) after the agronomic practices. For example, chambers were removed a day before slurry spreading and re-installed in the afternoon after spreading. During mineral fertilizer application, chambers were removed in the morning before application and re-installed in the afternoon.

**Table 1:** Application rates of cattle slurry and inorganic fertilizer during the cropping season

Date	Application	N-input (kg ha^− 1^)	P-input (kg ha^− 1^)	K-input (kg ha^− 1^)
14 May 2019	Cattle slurry	20.8	12	46
17 May 2019	Inorganic fertilizer	100^†^	85^¥^	205^‡^

Nutrient sources: Nitrogen; ^†^Nitram (Ammonium nitrate), Phosphorus; ^¥^ triple superphosphate (P_2_O_5_), Potassium^‡^ muriate of potash (K_2_O)

#### Treatments description

i) No-Buffer control: A downslope area of the maize plots with no-buffer strip at the base of the hydrologically-isolated slope.
ii) Grass Buffer: Novel grass buffer (*Festulolium loliaceum* cv. Prior). The grass was planted at the end of 2016 at a seeding rate of 5 kg ha^− 1^, the recommended seeding rate for the species in the Devon area.
iii) Woodland Buffer: Deciduous woodland. Six species, namely Pedunculate oak (*Quercus robur* L.), hazel (*Corylus avellana* L.), Hornbeam (*Carpinus betulus* L.), Small-leaved lime (*Tilia cordata* Mill.), Sweet chestnut (*Castanea sativa* Mill.) and Wych elm (*Ulmus glabra* Huds.) were planted in the woodland buffer strips. Five individual plants (each 40 cm in height and bare rooted) of each species were planted 1.6 m apart in rows 2 m apart in December 2016 in the 10 × 10 m area, with 1.5 m tall protection tubes to remove risk of browsing by wild herbivores (e.g., deer). Planting was done at a density of 3000 plants ha^− 1^, the recommended planting density for the Devon area.
iv) Willow Buffer: Bio-energy crop included five willow cultivars, namely Cheviot, Mourne, Hambleton, Endurance and Terra Nova (all *Salix* spp.) of which the first three were newly developed and the rest older cultivars. Whips of willow approximately 30 cm in length were inserted flush into the ground in May 2016 at a population of 200 plants per 10 m x 10 m area, the recommended planting density for willows in the Devon area.

At one month before planting of the different riparian buffer vegetation, each of the three buffer strip areas were sprayed with glyphosate herbicide to remove pre-existing grassland vegetation to enable better establishment of the planted deep rooting grass (*Festulolium loliaceum* cv. Prior), willow and woodland trees. The deep rooting grass buffer strips were also rotavated prior to seed broadcasting. Each of the buffer strips was composed of two parts – the lower slope area with a 2 m strip of natural grass, and the upslope area with a 10 m strip of treated and planted vegetation. The 2 m strip of natural grass is required for cross-compliance in England; farmers with watercourses must adhere to GAEC (Good Agricultural and Environmental Condition) rule 1, the establishment of buffer strips along watercourses (DEFRA 2019). The 10 m x 10 m area (10 m width) is the GAEC recommended N fertilizer application limit away from surface waters.

### Field measurements and laboratory analyses

#### Greenhouse gas monitoring

##### Field sampling and analyses

Soil N_2_O and CH_4_ fluxes were measured using the static chamber (non-vented) technique (Chadwick et al. 2014; Charteris et al. 2020; De Klein and Harvey 2012). The opaque polyvinyl chloride chambers were square frames with lids (40 cm width x 40 cm length x 25 cm height) with an internal base area of 0.16 m^2^. Thirty-three chamber collars were inserted to a depth of 5 cm below the soil surface using a steel base, and installation points were marked using a hand-held GPS so that they could be moved into the same positions after periodic removal for agronomic activities (e.g., tillage). In the willow and woodland riparian buffers, maize cropped areas, and no-buffer control, chambers were installed in-between two crop rows. In the grass riparian buffers, chambers were installed in pre-determined positions (Dlamini et al. 2022). At the beginning of the experiment, a gas sampling plan was developed with biweekly samplings after fertilizer application and less frequently (i.e., once or twice a month) afterwards (Dlamini et al. 2022), making a total of 16 measurement events. Gas sampling was conducted periodically from May to October 2019, between 10:00 and 13:00, using 60 mL syringes and pre-evacuated 22 ml vials fitted with butyl rubber septa. All chambers except for linearity chambers were sampled terminally at 40 min after closure (Chadwick et al. 2014). At each occasion, samples were collected at four-time intervals (0, 20, 40, and 60 min) from three chambers (called linearity chambers) to account for the non-linear increase in gas concentration with deployment time (Grandy et al. 2006; Kaiser et al. 1996). The quality of a calculated flux was calculated flux was adequately assessed using the goodness of fit test and/or by visual inspection; plateauing of gas concentration over time, and data that failed to meet the linearity standards were rejected (Collier et al. 2014). Additionally, ten ambient gas samples were collected adjacent to the experimental area with five at the start and five at the end of each sampling event. A Perkin Elmer Clarus 500 gas chromatograph (Perkin Elmer Instruments, Beaconsfield, UK) fitted with a Turbomatrix 110 automated headspace sampler with an electron capture detector (ECD) set at 300^o^C was used for N_2_O analysis and a flame ionization detector (FID) was used for CH_4_ analysis, after applying a 5-standard linear regression calibration. Separation was achieved by Perkin Elmer Elite-PLOT megabore capillary column, 30 m long and 0.53 mm Column Inside Diameter (ID), maintained at 35^o^C; N_2_ was used as a carrier gas (Cardenas et al. 2016).

##### Gas flux determination and GWP calculations

As suggested by Conen and Smith (2000), soil N_2_O and CH_4_ fluxes were calculated with the rate of change in concentration (ppm) within the chamber, which was estimated as the slope of a linear regression between concentration and chamber closure time. Cumulative N_2_O and CH_4_ fluxes were estimated by calculating the area under the gas flux curve after linear interpolation between sampling points (Mosier et al. 1996). The GWP of CH_4_ and N_2_O are respectively 28 and 310 times that of CO_2_ (IPCC 2014). Therefore, GWP was estimated by multiplying total CH_4_ and N_2_O fluxes by 28, and 310, respectively (Del Grosso et al. 2008).

#### Soil analyses and meteorological variables

Soil pH was measured with a pH meter (Jenway, Stafforshire, UK) using a soil suspension (1:2.5 soil:water ratio), and soil organic matter (OM) was determined using the loss-on-ignition (LOI) technique (Wilke 2005). Composite soil samples (0–10 cm), made up of four random sub-samples, were collected monthly within 1 m of each chamber using a soil corer with a semi-cylindrical gouge auger (2–3 cm diameter) (Poulton et al. 2018). Total oxidized N (TO-N) [nitrite (NO_2_^−^) and nitrate (NO_3_^−^)] and ammonium N (NH_4_^+^) were quantified by extracting field-moist 20 g soil samples using 2 M KCl and a 1:5 soil: extractant ratio; analysis was performed using an Aquakem™ analyser (Thermo Fisher Scientific, Finland). At every gas-sampling occasion, composite soil samples (0–10 cm) made of four random sub-samples were collected within 1 m from each chamber using a soil corer for gravimetric soil moisture determination. Dry bulk density (BD) was determined at the start of the experiment next to each chamber using the core-cutter method (Amirinejad et al. 2011) and used to convert the gravimetric moisture determined during each of the gas sampling events into percent soil water-filled pore spaces (WFPS). Daily precipitation was obtained from a nearby weather station within the Environment Change Network (ECN) at Rowden, North Wyke (Lane 1997; Rennie et al. 2020).

### Data processing and statistical analysis

Linear mixed models in Genstat 20 (VSN International, Hemel Hempstead, United Kingdom) were used to determine whether cumulative N_2_O, and CH_4_ differed with treatment. The random structure of each model (accounting for the experiment structure) was *block/plot/chamber*. The fixed structure (accounting for treatment effects) was treatment type/(treatment*distance). This model gave the following four tests in the output: (i) *Treatment type* – tested main maize cropped area vs. no-buffer control vs. riparian buffers, (ii) *Treatment type. treatment* – tested for differences between grass, willow, and woodland riparian buffers, (iii) *Treatment type. buffer distance* – tested for the difference between upper and lower riparian buffer areas, and (iv) *Treatment type. treatment. buffer distance* – tested for interaction between riparian buffer type and distance. A transformation was required to satisfy the equal variance assumption of the analysis of N_2_O. Due to the large negative values present for N_2_O, a modified square root transformation was used, *SIGN (N*_*2*_O)*√ (abs (N_*2*_*O)).* No transformation was required for the analysis of CH_4_.

Linear mixed models with the same random and fixed structures as those used for N_2_O, and CH_4_ were used to determine whether any measured soil variables (BD, pH, NH_4_^+^, TO-N, WFPS, and OM) differed with treatment. Pearson’s correlation coefficient (r) was used to evaluate the strength of relationships between soil and environmental factors and N_2_O/CH_4_ emissions. If linear mixed models indicated that treatment differences were present, least significant differences (LSD) were calculated to determine which specific treatment pairs resulted in the significant differences in N_2_O/CH_4_ emissions. All graphs were generated using Sigma Plot (Systat Software Inc., CA, USA).

## Results

### Meteorological and soil characteristics

#### Rainfall patterns

**Fig. 1:**
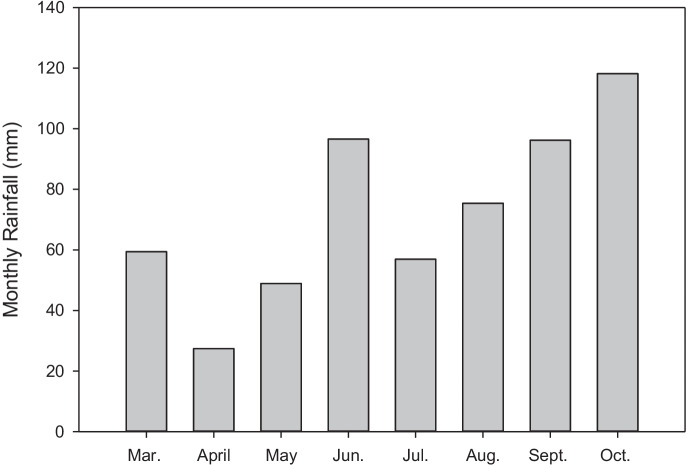
Total monthly rainfall during the experimental period

The total rainfall for the experimental period was 492.2 mm, and the highest rainfall event of 118.2 mm fell in October 2019. Before the highest rainfall in October, the second-highest rainfall events of 96.6 and 96.2 mm were recorded in June and September 2019, respectively (Fig. 1).

#### Soil variables

Soil pH ranged from 5.1 ± 0.17 and 5.5 ± 0.17, with the highest pH of 5.5 ± 0.17 from the willow riparian buffer, which was not significantly (*LSD = 0.29*) different from the grass or woodland riparian buffers. The largest soil BD of 1.2 ± 0.05 g cm^− 3^ was recorded in the no-buffer control, which was not significantly different from the upslope maize and the different vegetated riparian buffers (*LSD = 0.19*). Soil OM ranged from 9.0 (± 3.2) to 17.8 (± 2.3)%, with the largest %OM of 17.8 ± 2.3% recorded in the willow riparian buffer, which was not significantly (*LSD = 8.6*) different to the woodland riparian buffer (15.98 ± 2.3%). Soil OM in the vegetated riparian buffer strips was different from the upslope maize, but not from the no-buffer control, which was not different from the upslope maize (Tables 2 and 3).

**Table 2:** Summary of soil parameters (mean ± standard error) in the upslope maize and downslope riparian buffers with different vegetation (upslope maize: *n* = 12, no-buffer control: *n* = 3 and each riparian buffer: *n* = 6) before the commencement of the experiments in May 2019

Parameter	Upslope maize	No-buffer control	Grass buffer	Willow buffer	Woodland buffer	LSD
Soil pH	5.1 ± 0.17	5.1 ± 0.19	5.4 ± 0.17	5.5 ± 0.17	5.4 ± 0.17	0.29
Bulk density (g cm^− 3^)	1.21 ± 0.03	1.21 ± 0.05	1.1 ± 0.04	1.2 ± 0.04	1.2 ± 0.04	0.19
Organic matter (% w/w)	9.9 ± 1.3	9.0 ± 3.2	12.2 ± 2.3	17.8 ± 2.3	16.0 ± 2.3	8.6
NH_4_^+^-N (mg kg^− 1^ dry soil)	27.4 ± 2.98	20.6 ± 4.6	6.4 ± 2.7	13.6 ± 2.7	9.1 ± 2.7	7.8
TO-N^†^ (mg kg^− 1^ dry soil)	55.7 ± 1.7	42.8 ± 3.7	13.6 ± 3.0	4.99 ± 3.0	10.9 ± 3.0	10.0
WFPS^¥^ (%)	86.9 ± 5.3	81.7 ± 9.9	86.7 ± 7.2	102.9 ± 7.2	98.2 ± 7.2	18.6

^†^TO-N: total oxidized N; ^¥^WFPS: water-filled pore spaces

**Table 3:** P-values from linear mixed model results for each of the measured soil variables

Factors and interactions	OM	BD	NH_4_-N	pH	TO-N	WFPS
Area	0.04	0.29	< 0.001	< 0.001	< 0.001	0.23
Area * Treatment crop	0.31	0.13	0.16	0.238	0.173	0.24
Area * Buffer area	0.551	1	0.97	0.959	0.349	0.9
Area * Treatment crop * Buffer area	0.079	1	0.77	0.05	0.5	0.84

#### Soil mineral N-dynamics

**Fig. 2:**
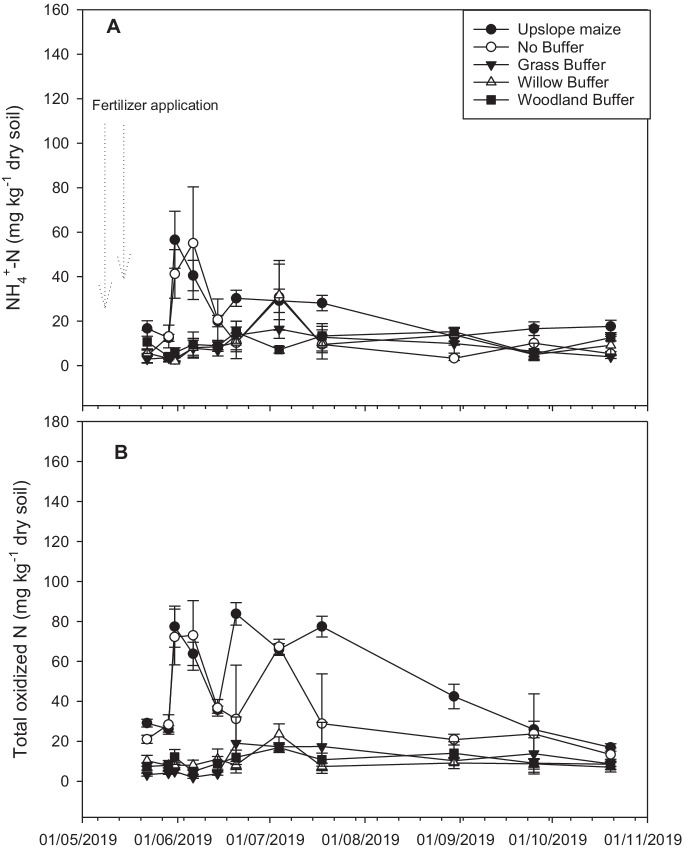
Soil NH_4_^+^ and total oxidized N (TO-N) in the upslope maize and downslope riparian buffers during the experimental period

At the commencement of the experiment, NH_4_^+^-N was < 17 mg kg^− 1^ dry soil in all of the treatments, with the largest of 16.7 ± 3.5 mg kg^− 1^ dry soil observed in the upslope maize. However, after the second sampling event, which had been preceded by two fertilizer application events (Table 1), NH_4_^+^-N increased by almost 3-fold in the no-buffer control and upslope maize treatments, but remained relatively low in the vegetated riparian buffers. Despite the high NH_4_^+^-N values in the no-buffer control and upslope maize crop areas after fertilization, values dropped to < 30 mg kg^− 1^ dry soil after the fourth sampling event and remained low until the end of the experimental period (Fig. 2). The average NH_4_^+^-N for the experimental period ranged from 6.4 ± 2.78 to 27.4 ± 2.8 mg kg^− 1^ dry soil, with the largest value of 27.4 ± 2.8 mg kg^− 1^ dry soil obtained from the upslope maize crop areas, which was not significantly (*LSD = 7.8*) different to the no-buffer control. It was, however, significantly different (*LSD = 7.8*) to the vegetated riparian buffers. Soil NH_4_^+^-N also differed between areas, but there was no evidence of any other differences between treatments. The NH_4_^+^-N in the vegetated riparian buffer strips was different from the upslope maize and no-buffer control, and the upslope maize and no-buffer control were not different from each other (Tables 2 and 3).

Total oxidized N was < 30 mg kg^− 1^ dry soil in all treatments at the commencement of the experiment (Fig. 2). However, after the second sampling event, TO-N increased 4-fold in the upslope maize and no-buffer control, but remained low in the riparian buffers. Despite a drop to ~ 35 mg kg^− 1^ dry soil in all of the upslope maize and no-buffer control areas during the fifth sampling event, the upslope maize emerged with the highest TO-N of ~ 81 mg kg^− 1^ dry soil during the sixth sampling event. However, these values dropped gradually up until the end of the experiment. Average TO-N for the experimental period ranged from 4.99 ± 3.0 to 55.7 ± 1.7 mg kg^− 1^ dry soil, with the highest value of 55.7 ± 1.7 mg kg^− 1^ dry soil obtained from the upslope maize. This was significantly different (*LSD = 10.0*) to all other treatments, except for the no-buffer control (Table 2).

#### %WFPS

**Fig. 3:**
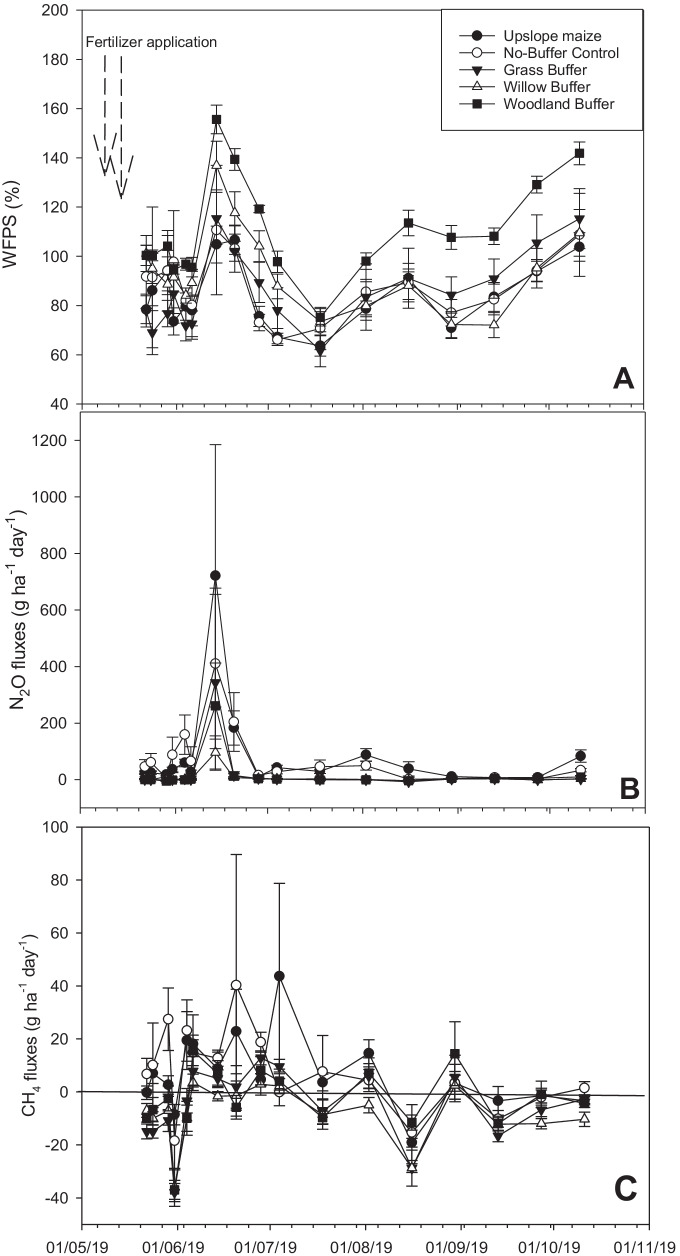
Daily (**A**) soil water filled pore space (WFPS), (**B**) N_2_O, and (**C**) CH_4_ fluxes in the upslope maize and downslope riparian buffers. Data points and error bars represent the treatment means for cropland (*n* = 12), no-buffer control (*n* = 3), grass, woodland and willow buffer (*n* = 6 for each) and standard errors respectively, during each sampling day. The vertical line in CH_4_ marks 0 fluxes

The highest %WFPS was observed during the fifth sampling event, with the overall highest estimate observed in the woodland riparian buffer treatment. The woodland riparian buffer maintained higher %WFPS values than the rest of the treatments during the experiment. The average %WFPS for the experimental period ranged from 81.7 (± 9.9) to 102.9 (± 7.2)%, with the highest value recorded in the willow riparian buffer, which was not significantly (*LSD = 18.6*) different to the woodland riparian buffer treatment, or any of the other treatments (Fig. 3A and Table 2).

### Gas emissions

#### Gas fluxes

##### Nitrous oxide

Nitrous oxide fluxes measured during each sampling event ranged between − 2.76 ± 1.98 g N_2_O ha^− 1^ day^− 1^ (willow riparian buffer) and 721.1 ± 464.3 g N_2_O ha^− 1^ day^− 1^ (upslope maize) and are shown in Fig. 3(B). The commencement of the experiment was marked by relatively low fluxes in all treatments. The low fluxes were immediately followed by the highest peak in all treatments, observed instantly after fertilizer application, with the maximum mean flux of 721.1 ± 464.3 g N_2_O ha^− 1^ day^− 1^ observed in upslope maize. There was also a smaller peak of 204 ± 5.7 g N_2_O ha^− 1^ day^− 1^ in the upslope maize at around the 1st of August 2019. After that fluxes remained < 10 g N_2_O ha^− 1^ day^− 1^ in all the treatments, with the upslope maize and no-buffer control maintaining predominantly higher fluxes until the end of the experiment.

##### Methane

Daily CH_4_ fluxes, which were mostly positive and sometimes negative, ranged between − 37.95 ± 3.43 and 67.45 ± 49.37 g CH_4_ ha^− 1^ day^− 1^ and are illustrated in Fig. 3(C). Similar to N_2_O fluxes, the commencement of the experiment was marked by low CH_4_ fluxes, which increased up to ~ 40 g CH_4_ ha^− 1^ day^− 1^ (in the upslope maize and no-buffer control) immediately after fertilizer application. After these peaks, CH_4_ fluxes remained low and mostly negative in all the treatments until the end of the experiment.

#### Cumulative gas emissions

##### Nitrous oxide

**Fig. 4:**
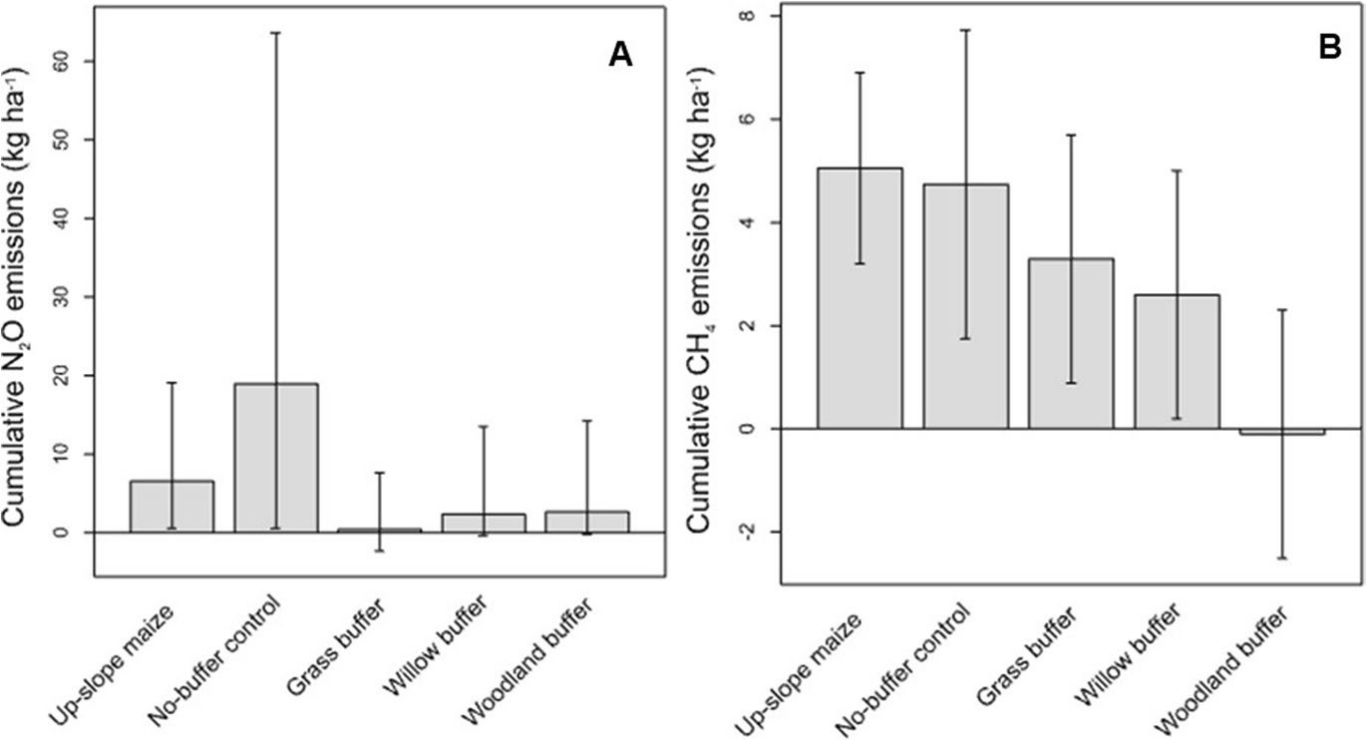
Cumulative (**A**) N_2_O and (**B**) CH_4_ emissions for the experimental period from the upslope maize and different downslope buffer vegetation. Error bars represent 95% confidence intervals for cropland (*n* = 12), no-buffer control (*n* = 3), grass, woodland and willow buffer (*n* = 6 each). Vertical lines are 95% confidence intervals

There was no evidence of significant treatment differences in N_2_O emissions between the upslope maize, no-buffer control and the three vegetated riparian buffers (*p* = 0.67) (Fig. 4A). Cumulative N_2_O emissions in descending order were no-buffer control 18.9 kg ha^− 1^ (95% CI: 0.5–63.6 ) > upslope maize; 6.5 kg ha^− 1^ (95% CI: 0.55–19.1 ) > woodland riparian buffer; 2.6 kg ha^− 1^ (95% CI: -0.27–14.2 ), willow riparian buffer; 2.3 kg ha^− 1^ (95% CI: -0.38–13.5) > grass buffer 0.38 kg ha^− 1^ (95% CI: -2.3–7.5).

##### Methane

The upslope maize and the no-buffer control (not significantly different from each other) emitted significantly higher cumulative soil CH_4_ fluxes than the three vegetated riparian buffers (*p* = 0.02) (Fig. 4B). Cumulative soil CH_4_ fluxes were in the descending order of upslope maize (5.1 ± 0.88 kg ha^− 1^) > no-buffer control (4.7 ± 1.4 kg ha^− 1^) > grass riparian buffer (3.3 ± 1.1 kg ha^− 1^) > willow riparian buffer (2.6 ± 1.1 kg ha^− 1^) > wood riparian buffer (-0.1 ± 1.1 kg ha^− 1^).

##### Global warming potential

Soil N_2_O-based GWP ranged from 1.2 ± 0.4 (willow riparian buffer) to 10.2 ± 4.7 (no buffer control) Mg CO_2_-eq. ha^− 1^ year^− 1^ (Table 6). A significantly higher GWP was found in the no-buffer control, which was not significantly different from the upslope maize. Soil CH_4_-based GWP ranged from 0.003 ± 0.36 (woodland riparian buffer) to 0.3 ± 0.03 (no buffer control) Mg CO_2_-eq. ha^− 1^ year^− 1^. Despite the large GWP found in the no buffer control, it was not significantly different to the other treatments, but to the woodland riparian buffer (Table 6).

##### Relationships between gas emissions and soil variables

**Fig. 5:**
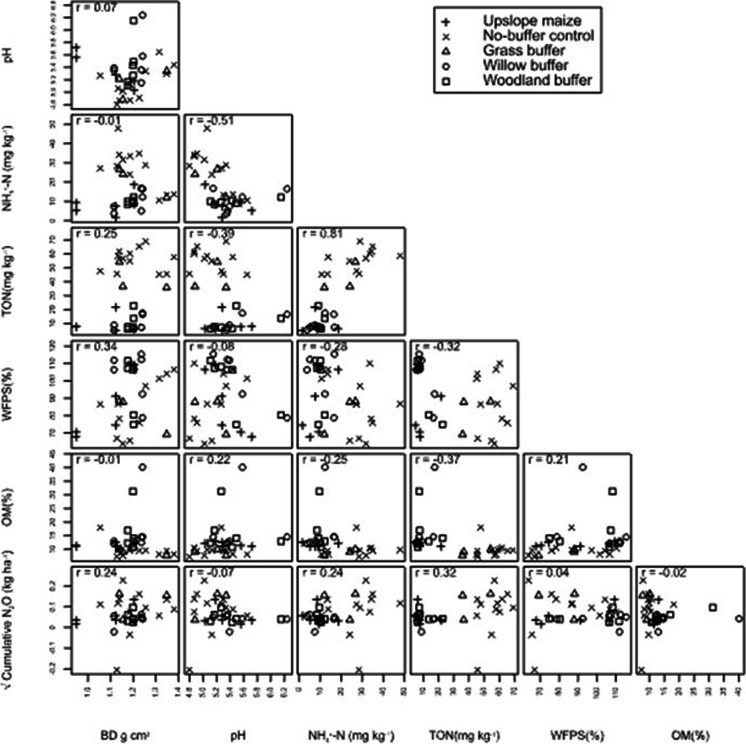
Scatterplot showing the relationships between the variables pH, soil NH_4_^+^-N, soil total oxized N (TO-N), water filled pore space (WFPS%), organic matter (OM), bulk density (BD) and cumulative N_2_O emissions for the upslope maize and the downslope riparian buffers with different vegetation treatments. r = Pearson’s correlation coefficient

**Fig. 6:**
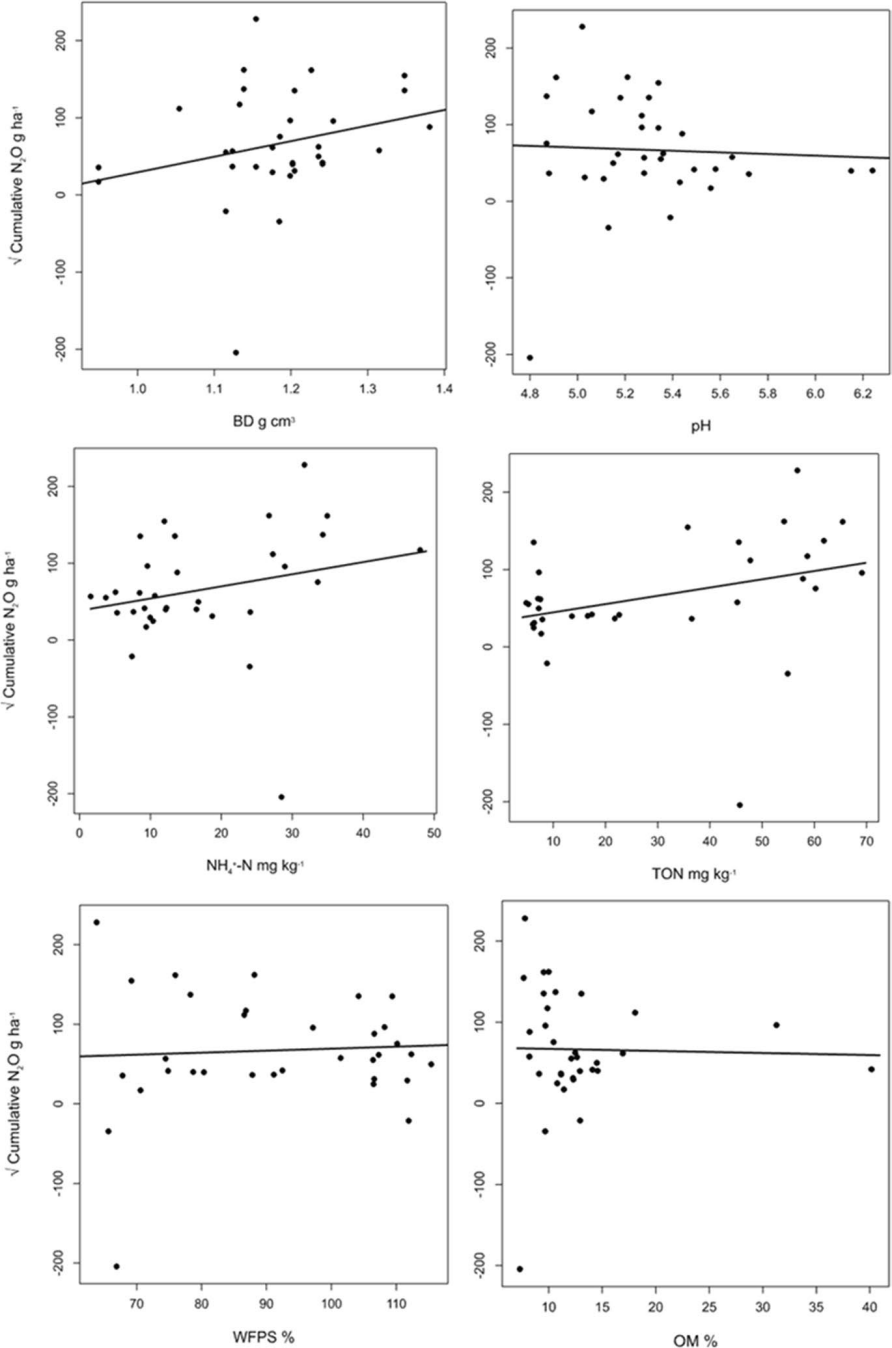
Relationships between cumulative N_2_O emissions and each of the soil variables

Table 4; Fig. 5 show that none of the soil variables had a significant relationship with cumulative N_2_O, but a slight relationship with TO-N (r = 0.32; *p = 0.065*). N_2_O emissions increased with an increase in soil BD, NH_4_^+^-N, TO-N, and %WFPS and decreased with an increase in pH and OM (Fig. 6).

**Table 4:** P-values for the slope of the fitted line in the N_2_O and soil variables model

Variable	Intercept	Standard error intercept	Slope	Standard error slope	*P-value*
BD	-172.6	142.1	201.9	119.98	0.126
pH	122.9	191.9	-10.56	36.194	0.786
NH_4_	38.29	23.48	1.58	1.1513	0.18
TO-N	33.97	18.18	1.068	0.555	0.065
WFPS	44.16	69.45	0.2518	0.75597	0.742
OM	69.7	29.76	-0.2556	2.05029	0.902

**Fig. 7:**
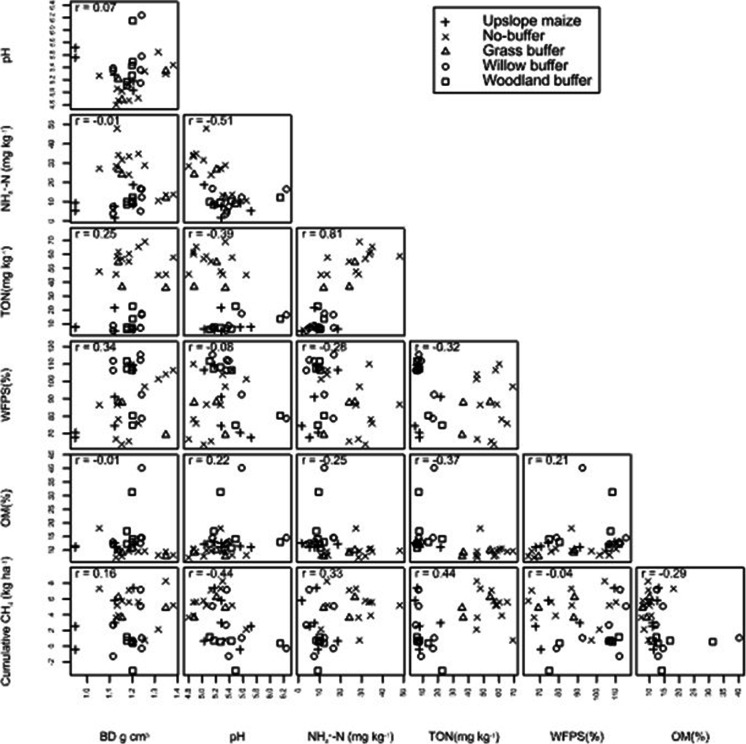
Scatterplot showing the relationships between the variables pH, soil NH_4_^+^-N, soil total oxidized N (TO-N), water filled pore space (WFPS%), organic matter (OM), bulk density (BD) and cumulative CH_4_ emissions for the upslope maize and the downslope riparian buffers with different vegetation treatments. r = Pearson’s correlation coefficient

**Fig. 8:**
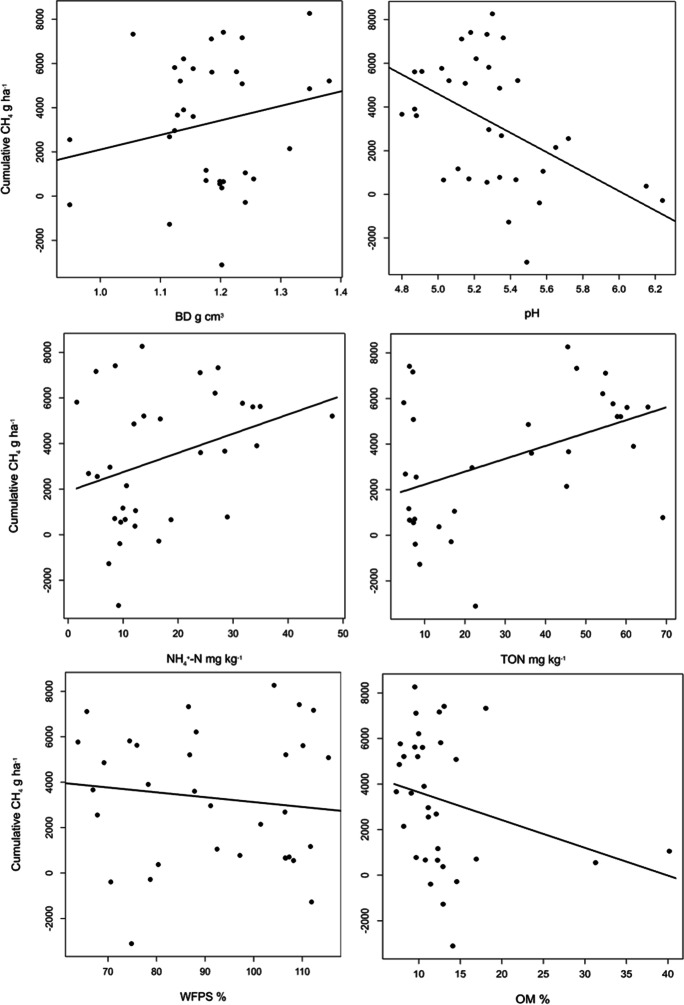
Relationships between cumulative CH_4_ emissions and each of the soil variables

Table 5; Fig. 7 show that pH (r = -0.44; *p* = 0.042) (perfect linear relationship and negative association), TO-N (r = 0.44; *p* = 0.005) (perfect linear relationship and positive association), and NH_4_^+^-N (r = 0.33; *p* = 0.056) (perfect linear relationship and positive association) had significant relationships with cumulative CH_4_ emissions. Soil CH_4_ emissions increased with increased BD, NH_4_^+^-N, and TO-N and decreased with an increase in pH, %WFPS, and OM (Fig. 8, Table 6).

**Table 5:** P-values for the slope of the fitted line in the CH_4_ and soil variables model

Variable	Intercept	Standard error intercept	Slope	Standard error slope	*P-value*
BD	-4469	6524	6575	5467.2	0.24
pH	26,829	5813	-4447	1094.7	0.042
NH_4_^+^	1901	918.6	84.33	42.303	0.056
TO-N	1663	925.3	56.41	18.574	0.005
WFPS	5265	2916	-21.41	30.548	0.489
OM)	4861	1197	-122	74.55	0.113

**Table 6:** Land-use (sample mean ± standard error) for upslope maize (*n* = 12), no-buffer control (*n* = 3) and each riparian buffer (*n* = 6) effects on global warming potential (GWP)

Land-use	GWP (Mg CO_2_-C equivalent ha^− 1^ year^− 1^)	
	N_2_O	CH_4_
Upslope maize	6.2 ± 3.5 ab^¥^	0.3 ± 0.03 a
No-buffer control	10.2 ± 4.7 a	0.3 ± 0.04 a
Grass buffer	2.5 ± 1.7 bc	0.2 ± 0.07 a
Willow buffer	1.2 ± 0.4 c	0.1 ± 0.07 ab
Woodland buffer	1.8 ± 800.5 bc	0.003.4 ± 0.04 b

^¥^Values within a column for each treatment followed by the same letter are not significantly different at the α = 0.05 probability level

## Discussion

### Gas emissions

#### Soil and environmental controls of gas fluxes

##### Nitrous oxide

The largest peak N_2_O flux observed in the upslope maize coincided largest %WFPS in the treatment. Large peaks also followed N fertilizer application events in the upslope maize and no buffer control (Fig. 3A and B). N_2_O fluxes following N fertilizer application are known to increase with increasing soil water content; most rapidly above 70% WFPS, wherein denitrification is a dominant process (Abbasi and Adams 2000; Dobbie et al. 1999; Granli and Bockman 1994; Skiba and Ball 2002). Soil moisture is one of the major drivers of N_2_O production, and directly affects production and consumption by influencing N-substrate availability, soil aeration, and metabolic activity of N_2_O-producing microorganisms, all of which control the capacity of soil to produce N_2_O (Di et al. 2014; Khalil and Baggs 2005; Simona et al. 2004). Nitrogen fertilizer has been reported as the main substrate for N_2_O-producing processes including nitrification and denitrification (Butterbach-Bahl et al. 2013; Dobbie et al. 1999). Thus, the higher fluxes were expected after N fertilizer application in the no-buffer control and the upslope maize (Table 1; Fig. 2) in the current study. Similarly, Halvorson et al. (2008) and Van Groenigen et al. (2004) reported that soil N_2_O emissions increased linearly with increasing N fertilizer. Additionally, there was an increase in N_2_O emissions with every increase in soil TO-N and NH_4_^+^-N (Fig. 6), which is in agreement with Mosier (1994), Mosier et al. (1996), and Barton and Schipper (2001). Notably, the woodland and willow riparian buffers had the highest %WFPS, but were characterised by lower N_2_O emissions during the peak flux. Not only the low N substrate due to unfertilized riparian buffers, but the reduced diffusion in the high soil moisture, caused a further reduction of N_2_O to N_2_ (Balaine et al. 2013; Hamonts et al. 2013). The no-buffer control and upslope maize had larger fluxes, which highlighted the interactive role of soil moisture and mineral N in enhancing N_2_O production (Klemedtsson et al. 1988).

The phenomenon of negative N_2_O fluxes is well documented to be dominant when high soil moisture (%WFPS) coincide with low mineral N (Chapuis-Lardy et al. 2007; La Montagne et al. 2003). Previous studies concur with the current study, for instance, in the first five events for the woodland and two events for grass riparian buffer, we observed negative N_2_O fluxes. The negative N_2_O fluxes coincided with high %WFPS and low mineral N (since riparian buffers were not directly fertilized) in the aforementioned treatments, which confirms the findings of other studies.

The larger N_2_O fluxes, coinciding with higher soil moisture (Fig. 3A and B) in all the treatments after the third sampling event, may have been due to higher N mineralisation potential. Higher mineralisation is known to increase under water saturated conditions and hampered by low soil moisture (Hackl et al. 2004). The larger N_2_O flux in the upslope maize and no-buffer control meant that the two treatments had higher N mineralisation potential compared to vegetated riparian buffers. It has been previously reported that differences in soil (Reich et al. 1997) and vegetation (Priha and Smolander 1999) characteristics significantly influence N mineralisation. Higher soil pH levels are also known to render conditions favourable for N mineralisation (Hackl et al. 2004). However, in our study, the vegetated riparian buffers with higher soil pH values had lower N_2_O fluxes compared to the no-buffer control and upslope maize, which had low pH values but maintained high N_2_O fluxes. This meant that other factors influenced N mineralisation more than high soil pH, but was not confirmed in the current study.

##### Methane

The overall positive CH_4_ emissions from all treatments was likely the result of the high %WFPS experienced during most of the experimental period. The upper values (~ 5 kg CH_4_ ha^− 1^) are similar to those reported by Groh et al. (2015). Field investigations have identified soil water content as one of the critical controls of CH_4_ production and consumption in soils from different ecosystems (Khalil and Baggs 2005; Kim et al. 2010; Wu et al. 2010). High soil moisture contents are documented drivers of CH_4_ production and emissions in soils; as a group of strictly anaerobic bacteria produce the majority of CH_4_ in reduced environments (Ehhalt et al. 2001; Ehhalt and Schmidt 1978; Yang and Chang 1998). Similar to other studies, our study recorded peak CH_4_ fluxes immediately after the highest %WFPS occurred (Fig. 3A and C). Soil moisture directly affects the capacity of soil to produce or consume CH_4_ through its influence on C-substrate availability, soil aeration, and metabolic activity of CH_4_ producing microorganisms (Khalil and Baggs 2005; Simona et al. 2004). The role of soil moisture in CH_4_ production and subsequent emissions was verified by the low (sometimes negative) CH_4_ fluxes, coinciding with low soil %WFPS at the end of August (Fig. 3A). Similarly, Luo et al., (2013) observed that soil moisture affected soil CH_4_ consumption through its effect on substrate availability and redistribution, soil aeration, and the metabolic activity of microorganisms. In October 2019, low soil CH_4_ fluxes were observed in all treatments despite the high %WFPS (Fig. 3A and C). We speculate that soil C for CH_4_-producing processes including mineralisation, may have been exhausted during this time, as was observed by Yu et al. (2013), but we did not verify this in the current study.

#### Gas emissions in upslope maize and downslope riparian buffer strips

##### Nitrous oxide

For a riparian buffer to be considered a threat to pollution swopping between air and water, it must emit more N_2_O than the cropland it serves (Fisher et al. 2014). In the current study, the no-buffer control proved to be an atmospheric concern, since it generated the highest N_2_O emissions compared to the upslope maize and the three vegetated riparian buffers (Fig. 4A). Despite the large N_2_O emissions in the no-buffer control, they were not significantly different to the vegetated riparian buffers and the upslope maize. The findings were similar to Baskerville et al. (2021) and De Carlo et al. (2019), who observed no significant differences in N_2_O emissions amongst the riparian zones. Baskerville et al., (2021) also reported that there were no significant differences when comparing these zones to the agricultural land. The maximum cumulative emissions of 20 kg N_2_O (~ 12 kg N ha^− 1^) were similar to Kim et al. (2009) (2-year study) and Groh et al. (2015) (1-year study), who observed 24 and 14.8 kg N_2_O ha^− 1^, respectively, in maize in a Humid Continental climate. We acknowledge that the differences in N_2_O emissions between the current study and the previous studies may have been due to different histories and N fertilization, but we did not have a way to confirm this in the current study. The high N_2_O emissions in the no-buffer control could have been due to applied fertilizer N (particularly readily available inorganic N), which increased mineral N availability for the N_2_O-producing nitrification and denitrification processes. Similar findings were reported by Dobbie et al. (1999) and (Butterbach-Bahl et al. 2013). In fact, the high N_2_O emissions in the no-buffer control showed a downward movement of the fertilizer applied N with rainwater. This was further attested to by the high mineral N (TO-N and NH_4_^+^) in the no-buffer control compared to the remainder of the treatments (Table 2) and an increase in N_2_O emissions with every increase in mineral N (Fig. 6). The vegetated riparian buffers had low N_2_O emissions; which indicated that they served their purpose of intercepting and processing N to N_2_ through denitrification induced by their high soil moisture (Groffman et al. 1991; Knowles 1982) before off-site delivery. Interestingly, the riparian buffers had ideal conditions to promote full denitrification (conditions highlighted by Dlamini et al. 2020), reducing NO_3_^−^ to N_2_. Especially at the high moisture and in the case of willow and woodland, the high organic matter and potentially available C explained their low N_2_O compared to the upslope pasture and no buffer control. The low N_2_O emissions in the vegetated riparian buffers (Fig. 4A) could also have been because the riparian buffer strips were not directly fertilized. This highlighted the role of fertilizer N in increasing mineral N availability for N_2_O producing processes, as discussed by Davis et al. (2019), Hefting et al. (2003) and Iqbal et al. (2015). The second-highest N_2_O emissions observed in the upslope maize could have also been due to N fertilizer application.

The N_2_O emission factor is determined as the percentage of the fertilizer-induced N_2_O-N emission relative to N fertilization, where fertilizer-induced N_2_O-N emission is usually measured as the difference in N_2_O emission between fertilized and unfertilized soil under otherwise identical conditions (Wang et al. 2018). In our study, the N-fertilized upslope maize and the no-buffer control had emission factors of 3.25% and 13.6%, respectively. These were much higher than the default 1% proposed by the IPCC’s Tier-1 model for croplands, assuming a linear response to N fertilization (Eggleston et al. 2006). Despite the emission factors of the upslope maize and no-buffer control being much higher than the IPCC’s suggested 1%, the results of the current study were similar to those of Jungkunst et al. (2006), Kaiser et al. (1996), and Rudaz et al. (1999). These studies recorded N_2_O-emissions from agricultural soils as a result of applied fertilizer N to vary between 0.005% and 15.5%. Some authors, including Butterbach-Bahl et al. (2013), Rashti et al., (2015), Stehfest & Bouwman (2006), and Venterea et al. (2012) reported that N_2_O emissions are not only determined by external N fertilizer input, but also by responses controlled by key soil properties.

##### Methane

The fact that the upslope maize and no-buffer control treatments exhibited high CH_4_ emissions may have been a result of NH_4_^+^-N based fertilizer applied in the two treatments (Table 1; Figs. 2 and 4B). NH_4_^+^-N inhibits CH_4_ oxidation (Hütsch 1998; Kravchenko et al. 2002; Tlustos et al. 1998); which often results in a net increase in CH_4_ emitted from soil (Bronson and Mosier 1994). This inhibition is either a general salt effect (Gulledge and Schimel 1998) with a competition between ammonia (NH_3_) and CH_4_ for methane monooxygenase enzymes (Bédard and Knowles 1989), or non-competitive inhibition by hydroxylamine (NH_2_OH) or nitrite (NO_2_^−^) produced during NH_3_ oxidation (King and Schnell 1994). To further emphasize the role of mineral N in inhibiting CH_4_ oxidation, the three vegetated and unfertilized riparian buffers had significantly lower CH_4_ emissions than the upslope maize and the no-buffer control (Fig. 4B).

#### Global warming potentials

The high N_2_O and CH_4_-based GWP in the no buffer control showed that growing a maize crop without implementing riparian buffer vegetation may have increased the risk of GWP. On a positive note, implementing willow and woodland riparian buffers in tandem with a maize crop may reduce the risk of GWP while simultaneously contributing to their intended use to improve water quality.

### Implications of the findings

Our findings have a number of implications especially in research and environmental policy. Although riparian buffer strips are conventionally implemented in intensive farming practices to enhance water quality in the UK and elsewhere, our work demonstrates additional benefits regarding their uptake of gaseous emissions. Many countries have focused on the urgent need to tackle the climate emergency and robust evidence on the efficacy of interventions for reducing harmful gaseous emissions is critical for engaging stakeholders including farmers.

The findings have implications for calibration of process-based models to simulate N_2_O and CH_4_ emissions from croplands and/ or riparian buffer areas, which has been challenging due to lack of data availability. Process-based models including the Riparian Ecosystem Management Model (REMM) (Lowrance et al. 2000) have been calibrated to simulate soil processes under riparian buffers. For example, REMM has been used to simulate groundwater movement, water table depths, surface runoff and annual hydrological budgets (Inamdar et al. 1999b). The model has also been used to simulate N, phosphorus (P), and C cycling (Dukes and Evans 2003; Inamdar et al. 1999a) interactions between riparian buffer systems. Other watershed models, such as the Soil and Water Assessment Tool (SWAT), have been calibrated to assess the effectiveness of riparian buffers for reducing total organic N-losses in a watershed (Lee et al. 2020). A landscape model, the Morgan-Morgan-Finney topographic wetness index (MMF-TWI), has been calibrated to simulate erosion reduction using riparian buffers (Smith et al. 2018a). However, to the best of our knowledge, none of these mechanistic models have been calibrated to simulate N_2_O and CH_4_ emissions from riparian buffers and further compared with emissions from croplands. Even though process-based models (e.g., Denitrification-Decomposition: DNDC) have been calibrated to simulate biogeochemical cycles including N_2_O emissions from grass riparian buffers in Illinois, USA (Gopalakrishnan et al. 2012), to the best of our knowledge, this model has not been calibrated to simulate greenhouse gas emissions from riparian buffers in the UK.

### Limitations of the study

One of the significant limitations of the study was the use of a replicated plot-scale experimental facility. This meant that our results represented the climate, soil, and environmental conditions prevailing at the experimental site at North Wyke, Devon, UK. Similar conditions in terms of annual rainfall, soil and farming system, are present in 1843 km^2^ of farmed land across England (Collins et al. 2021). Our results provide robust data on short-term N and C gaseous emissions and clearly, longer-term measurements would help in confirming our findings. Although the static chamber is cheap and easy to use, a possible shortcoming is that it was used to trap gas in the field for the experiment. For instance, Healy et al. (1996) and Rochette (2011) reported that insertion of chambers into the soil may limit lateral gas exchange. However, Rochette (2011) suggested that such limitations may be overcome by inserting chamber collars prior to use. Rochette (2011) also argued that this practice may affect soil temperature by shading the soil, soil moisture by preventing soil run-off, and gas exchange through formation of shrinkage cracks at the collar-soil interface.

## Conclusions

Our replicated plot-scale facility experiment showed that the N-fertilized no-buffer control and upslope areas used for maize cropping might be significant N_2_O and CH_4_ sources, respectively. Furthermore, the low N_2_O and CH_4_-based GWP from the willow and woodland riparian buffers show that willow may mitigate GWP when implemented for water quality protection purposes in maize production. Accordingly, our results attest to the unintended benefits of riparian buffers for reducing gaseous emissions, despite primarily being implemented as water quality protection measures.

## Data Availability

Data available from authors on request.
